# Blind trials of computer-assisted structure elucidation software

**DOI:** 10.1186/1758-2946-4-5

**Published:** 2012-02-09

**Authors:** Arvin Moser, Mikhail E Elyashberg, Antony J Williams, Kirill A Blinov, Joseph C DiMartino

**Affiliations:** 1 Advanced Chemistry Development, Toronto Department, 110 Yonge Street, 14th floor, Toronto, Ontario, M5C 1T4, Canada; 2 Advanced Chemistry Development, Moscow Department, 6 Akademik Bakulev Street, Moscow 117513, Russian Federation; 3 Royal Society of Chemistry, 904 Tamaras Circle, Wake Forest, NC, 27587, USA

## Abstract

**Background:**

One of the largest challenges in chemistry today remains that of efficiently mining through vast amounts of data in order to elucidate the chemical structure for an unknown compound. The elucidated candidate compound must be fully consistent with the data and any other competing candidates efficiently eliminated without doubt by using additional data if necessary. It has become increasingly necessary to incorporate an *in silico *structure generation and verification tool to facilitate this elucidation process. An effective structure elucidation software technology aims to mimic the skills of a human in interpreting the complex nature of spectral data while producing a solution within a reasonable amount of time. This type of software is known as computer-assisted structure elucidation or CASE software. A systematic trial of the ACD/Structure Elucidator CASE software was conducted over an extended period of time by analysing a set of single and double-blind trials submitted by a global audience of scientists. The purpose of the blind trials was to reduce subjective bias. Double-blind trials comprised of data where the candidate compound was unknown to both the submitting scientist and the analyst. The level of expertise of the submitting scientist ranged from novice to expert structure elucidation specialists with experience in pharmaceutical, industrial, government and academic environments.

**Results:**

Beginning in 2003, and for the following nine years, the algorithms and software technology contained within ACD/Structure Elucidator have been tested against 112 data sets; many of these were unique challenges. Of these challenges 9% were double-blind trials. The results of eighteen of the single-blind trials were investigated in detail and included problems of a diverse nature with many of the specific challenges associated with algorithmic structure elucidation such as deficiency in protons, structure symmetry, a large number of heteroatoms and poor quality spectral data.

**Conclusion:**

When applied to a complex set of blind trials, ACD/Structure Elucidator was shown to be a very useful tool in advancing the computer's contribution to elucidating a candidate structure from a set of spectral data (NMR and MS) for an unknown. The synergistic interaction between humans and computers can be highly beneficial in terms of less biased approaches to elucidation as well as dramatic improvements in speed and throughput. In those cases where multiple candidate structures exist, ACD/Structure Elucidator is equipped to validate the correct structure and eliminate inconsistent candidates. Full elucidation can generally be performed in less than two hours; this includes the average spectral data processing time and data input.

## Background

With the advances of high throughput data collection and data processing for a variety of analytical techniques (*e.g*. NMR, MS, IR), there is an increasingly higher demand on the chemists to promptly and efficiently elucidate the structure of unknowns [1,2]. This bottleneck has encouraged researchers to search for robust technologies that can improve throughput and ensure accuracy in solving the problem and computer-assisted structure elucidation (CASE) applications have been the primary area of focus [3-5]. The development of a CASE application mandates an adaptable application to a variety of challenges inherent with solving the complete structure for an unknown compound based on typical spectral data.

We present the results of a unique, multiyear worldwide blind trial study on a CASE application, namely ACD/Structure Elucidator (StrucEluc) [5,6], and the necessary evolution of the CASE technology as various complex challenges were encountered. StrucEluc is an artificial intelligence system that can interpret data from a variety of spectral datasets including 1D and 2D NMR, MS, IR, *etc*. Based on the restrictions imposed by the set of spectral data, all possible atomic combinations are worked out to ensure that no plausible candidate escapes consideration [7]. In addition, a general viewpoint is presented regarding the inherent trends in the complex nature of the data associated with each challenge.

## Results and discussion

### 1. Categorizing the Global Challenges

In 2003 a worldwide challenge [8] was initiated with the intent of testing and showcasing the performance of the CASE expert software system StrucEluc. Originally intended as a single-blind trial, a scientist was requested to submit spectral data for an organic compound while withholding the structural skeleton so as to not bias the operator of the software. The software would be used to generate one or more candidate structures consistent with the spectral data, the results would be reported to the scientist and they would confirm validity of the analysis.

As of January 2011, a total of 112 official challenges had been received from a variety of institutions including academic (50%), pharmaceutical (42%), industrial (5%) and government (3%) institutions. The global responses segmented into the following regions: North America at 47%, Europe at 30%, Asia at 18% and the remaining continents at 5%.

Each challenge provided a variety of degrees of complexity and expertise in the elucidation of unknown compounds. For ten of the 112 challenges (9%), the structures were unknown to both the submitter and the analyst and the double-blind trials were highly valued and scientifically interesting to both parties. In addition, a separate set of five challenges (4%) were submitted to ascertain and validate the submitter's proposed structure; this ensured that additional candidates were not overlooked.

**Figure 1:**
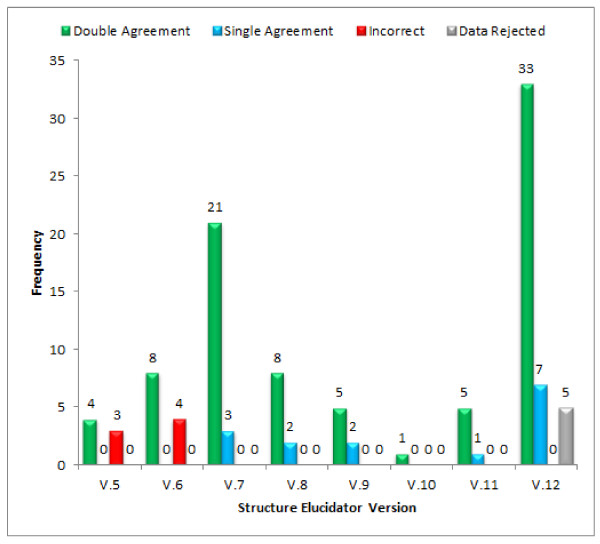
**A summary of the performance of the StrucEluc system on data for 112 blind trials submitted during the years 2003 - 2011**. The results represent challenges performed using an incremental version. For example, a total of 7 challenges were analyzed with version 5. For version 6, 12 new and different challenges were analyzed.

Figure 1 illustrates the evolution of the StrucEluc software in respect to the number of challenges received when the various incremental versions were available. The challenges were divided into four results categories: Double Agreement, Single Agreement, Incorrect and Data Rejected. The Double Agreement category (colored in green) indicates that the proposed structure was agreed upon and validated by both the submitter and software. This also includes the double-blind trials. The Single Agreement (colored in blue) indicates that the submitter was not confident enough to verify the most probable structure delivered by the program or did not respond back to confirm. In most of these challenges, this structure is considered proprietary and acknowledging its correctness with an outside source could breech company policies. A total of 100 challenges fell into one of these two categories.

For the Incorrect category (colored in red), the structure generated by the software was not in agreement with the proposed structure of the submitter. For StrucEluc version 5, three of the seven trials consisted of unknowns larger than 1000 Da, thus surpassing the size limitations of the software. This limitation in place at the time of the analysis has since been removed. The remaining four trials did not want to share their proposed candidates. For the last category, Data Rejected (colored in grey), the required data were inadequate for analysis due to poor instrument practice, exhibited extremely poor S/N or contained indiscernible artefacts or impurities, *etc*. A total of 12 challenges fell into one of these two categories.

The StrucEluc software failed to generate a structure corresponding to that expected by the submitter only with versions 5 and 6, released in 2003 and 2004 respectively. Reviewing the data showed that the software lacked several features that prevented the software from successfully elucidating the structures. This included library searches using chemical shifts and handling ambiguous assignments for COSY and HMBC correlations. The ongoing challenging of the system using hundreds of real problems helped to direct the development of the system as it is impossible to imagine all difficulties *a priori*. The limitations were discovered during the process of problem solving and the software was improved incrementally over time to overcome them.

As the StrucEluc software was developed to accommodate specific nuances associated with an elucidation, the number of submitted challenges also increased, together with the number of correct structures. The popularity of the challenge attracted the attention of a new group of chemists, specifically Ph.D. students, seeking out answers to structure elucidation problems that could be included into their thesis. For version 12, four out of the five challenges were submitted by students requiring assistance in their thesis work. Unfortunately, for one of the problems only a^ 13^C NMR spectrum was received and a library search resulted in no direct hits; the challenge proceeded no further as additional data was not made available. The remaining challenge was rejected due to poorly collected^ 1^H NMR,^ 13^C NMR and^ 1^H-^13^C HSQC spectra and inconsistencies among the data. In all five cases we offered guidance regarding how to collect better data but these particular challenges did not progress further. The submitters also declined to have their data showcased.

In one particular example the submitter presented twenty-four tabular^ 13^C chemical shifts with a molecular formula (MF) of C_20_H_w_N_x_O_y_S_z _where w, x, y and z are used to obscure the numerical values for the MF. No further clarification was made by the submitter despite a request for further information. This data was insufficient to proceed with an analysis, because in such cases the number of structures that can correspond to the available data is hardly constrained.

The incremental analyses and successes of the system were a means by which to build confidence in the general applicability of a CASE application to assist chemists. Each iterative development utilized new strategies to accommodate the diverse nature of the challenge data [9,10].

**Figure 2:**
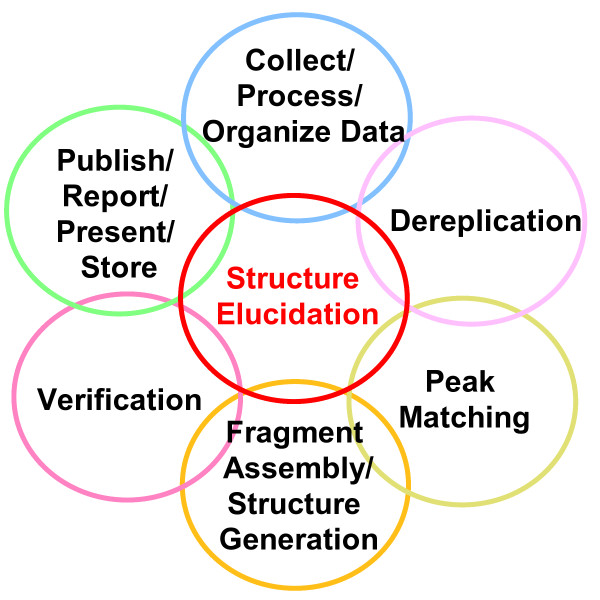
**An arrangement of the common tasks incorporated into a complete structure elucidation workflow **[11]. A structure elucidation encompasses several tasks including data collection, library searching and fragment assembly.

There are a number of factors that contribute to the successful elucidation of a structure using a CASE system. Experience has shown that time invested upfront offers an improved probability of a successful result. The amount of time invested in collecting a diverse range of data and of high enough signal-to-noise is important. Also, the care with which data is processed, the time invested in peak picking and the piecing together of fragments to complete a proposed structure(s) through a structure generation process all contribute to a successful result (Figure 2) [11]. When data under analysis present complicated and numerous possibilities to consider, then CASE systems present an alternative approach [3,12].

### 2. Data Processing and Dereplication

For the 112 challenges discussed in this publication, encompassing both Double and Single agreement analyses and listed in Table 1, the average processing time was determined to be around 84 minutes (~1.4 hours). This includes the time spent on processing the data (e.g. adjusting the window functions, Fourier transformation, phasing, peak picking, assessing impurities, *etc*.). After processing of the NMR data, dereplication is the first step and consumes only about 3 minutes. The spectral library used for dereplication comprises of more than 19,000,000 records with structure information and assigned^ 13^C chemical shift values. The computational time spent performing structure generation averages just over 25 minutes and, in this time period, an average of 2639 structures are generated by the software excluding duplicate structures with differing NMR assignments. It is necessary to keep in mind that the computational time and the number of candidate structures strongly depend on the uniqueness of the initial information. The input of additional data can reduce the computational time and the number of potential candidates quite dramatically. The generated candidates are ranked according to the deviation between the predicted^ 13^C chemical shifts and the experimental shifts so that the submitter can quickly assess the top candidates. There is a clear advantage of elucidating with a software tool over attempting an elucidation by hand as this ensures that every potential isomer is assessed.

**Table 1:** Ranges for the calculation times and structures generated across the challenges.

	Processing Time (min.)	Library Search Time (min.)*^a^*	Generation Time (min.)*^a^*	Structures Generated
**Minimum**	0 (Tabular)	1	1	1
**Maximum**	245	10	240	100000
**Average**	84	3	26	2639

*^a ^*The calculation was performed using desktop PCs operating at processor speeds of 200 MHz to 2 GHz. For example, structure calculations for version 6.0 were conducted on a Pentium III 1 GHz system equipped with 512 MB RAM and using the Microsoft Windows 2000 operating system.

In order to initiate a structure elucidation challenge a minimal set of data is required from the submitter. Additional data was willingly accepted (see the Experimental section for more details). In those cases where there may be sample limitations and experiments may take a long time to acquire, for example, a ^1^H-^13^C HMBC may take weeks to acquire [11], dereplication was nevertheless feasible.

Dereplication is a quick and effective pre-screening approach for the identification of an unknown compound. There are several advantages to searching across a database or library of known structures when a set of data is available. These include saving time, energy, instrument time and ultimately this of course equates to saving money. The ultimate goal is to determine whether a compound is novel or not. If a compound is not found in the database then dereplication can at least help to identify potential classes of chemical compounds similar to the unknown on the basis of the heuristic rule that "similar structures have similar spectra". In StrucEluc the searches can be performed with a MF, monoisotopic mass, or^ 13^C NMR chemical shifts.

Nine percent of the submitted challenges were solved simply with a library search through two available databases, an internal library of ~ 400,000 records and the PubChem library [13] at ~ 19,000,000 records for which chemical shifts were pre-calculated. The search process involved taking the^ 13^C chemical shifts from the 1D NMR data or extracting it from the 2D NMR data and searching for compounds matching the chemical shifts.

**Figure 3:**
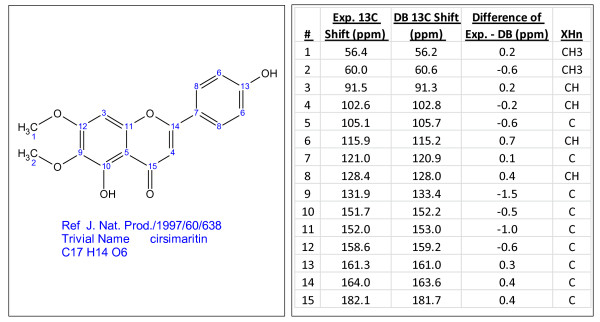
**Cirsimirtin (C_17_H_14_O_6_), a compound identified through dereplication using experimental^ 13^C chemical shifts**. The search was performed using the internal library.

**Figure 4:**
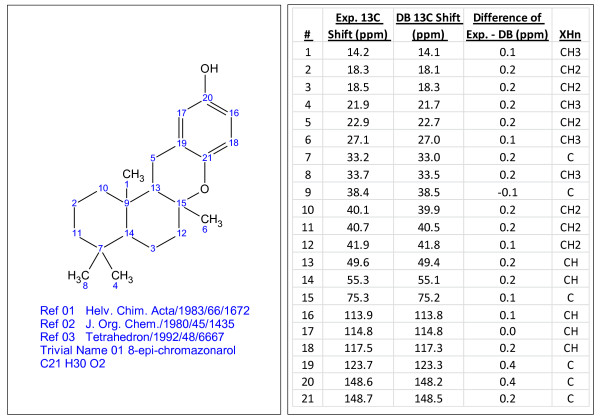
**8-epi-chromazonarol (C_21_H_30_O_2_), a compound identified through dereplication using experimental^ 13^C chemical shifts**. The search was performed using the internal library.

**Figure 5:**
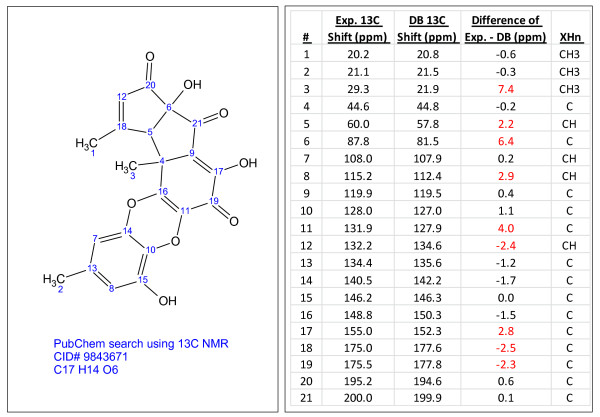
**CID#9843671 (C_17_H_14_O_6_), a compound identified through dereplication using experimental^ 13^C chemical shifts**. The search was performed using the PubChem library [13] containing^ 13^C chemical shifts predicted using ACD/CNMR Predictor [21]. Chemical shift differences greater than 2 ppm are highlighted in red.

**Figure 6:**
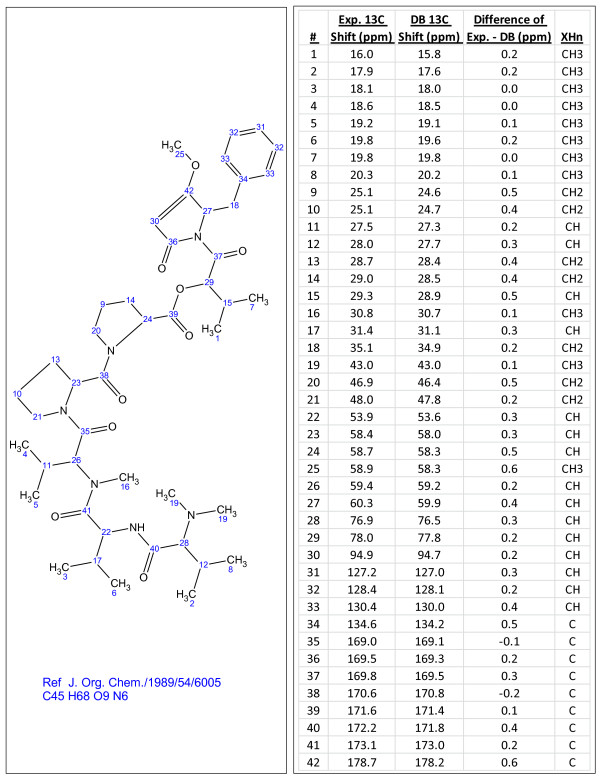
**C_45_H_68_N_5_O_9_, a compound identified through dereplication using experimental ^13^C chemical shifts**. The search was performed using the internal library.

**Figure 7:**
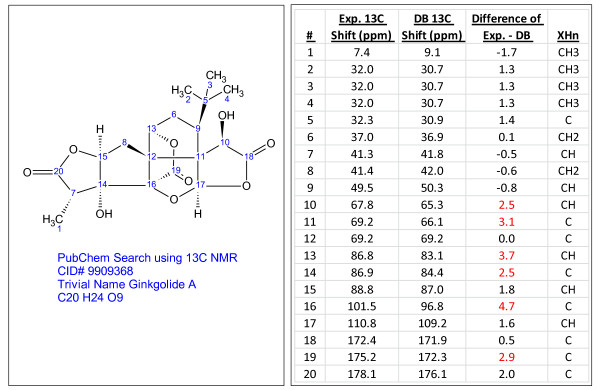
**Ginkgolide A (C_20_H_24_O_9_), a compound identified through dereplication using experimental^ 13^C chemical shifts**. The search was performed using the PubChem library [13] containing^ 13^C chemical shifts predicted using ACD/CNMR Predictor [21]. Chemical shift differences greater than 2 ppm are highlighted in red.

A series of random examples of compounds identified by searching the internal and PubChem libraries are presented in Figures 3, 4, 5, 6 and 7. The compounds vary in the degree of complexity, size and nature of the compound including synthetic and natural products. It should be noted that these searches consume very little time, only a few minutes.

**Figure 8:**
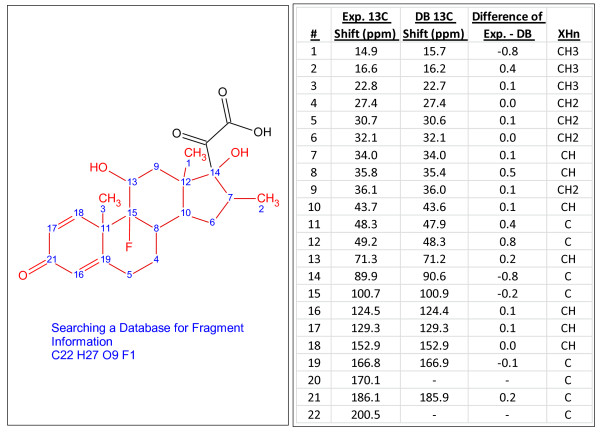
**C_22_H_27_O_9_F_1_, an example of a single-blind challenge elucidated using StrucEluc version 7**. The fragment, shown in red, was retrieved via a fragment-based dereplication using the internal fragment library. The^ 13^C chemical shifts are listed in the right panel.

In 3% of the challenges, an internal fragment library (~2,000,000 records) was searched and fragment information was utilized to complete the elucidation. Not all challenges were searched through the fragment library (*vide infra*). Figure 8 illustrates an example of a challenge where the fragment shown in red was found from a^ 13^C chemical shift search of the fragment library. Such a fragment dereplication approach can assist with the elucidation of novel compounds with similar scaffolds to known compounds and thus reduce time spent on structure generation.

### 3. Structure Generation

**Figure 9:**
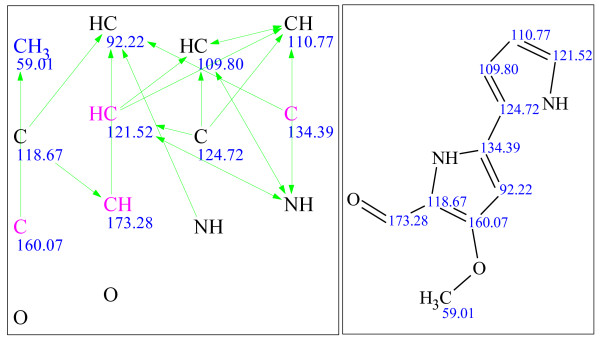
**Example 1: a single-blind challenge **[22]** elucidated using StrucEluc version 7**. The molecular connectivity diagram (MCD) in the left panel consolidates the data from the MF and the spectral data into a single diagram. The blue, green and black lines represent the connectivities extracted from a^ 1^H-^1^H COSY, ^1^H-^13^C HMBC spectra and user fragments, respectively. The dashed lines indicate ambiguity in the assignment of the correlations. The carbon atom colors dictate the hybridization state of the atom: blue, pink and black represent *sp^3^, sp^2 ^*and *sp/sp^2^/sp^3^*, respectively. The right panel exhibits the most probable candidate with assigned^ 13^C chemical shifts.

**Figure 10:**
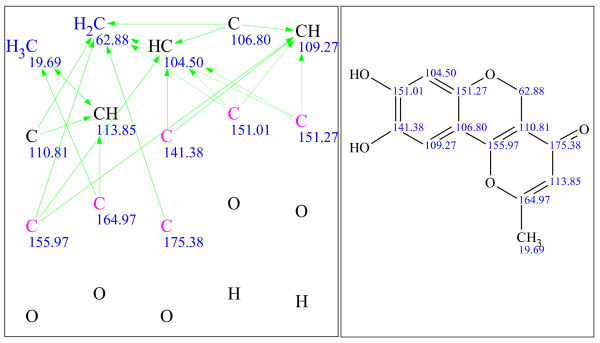
**Example 2: a single-blind challenge **[23]** elucidated using StrucEluc version 7**.

**Figure 11:**
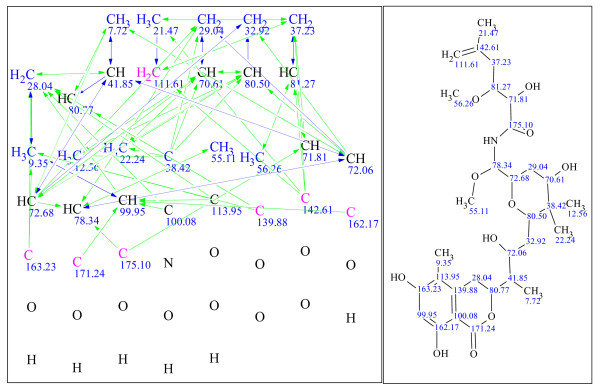
**Example 3: a single-blind challenge **[24]** elucidated using StrucEluc version 9**.

**Figure 12:**
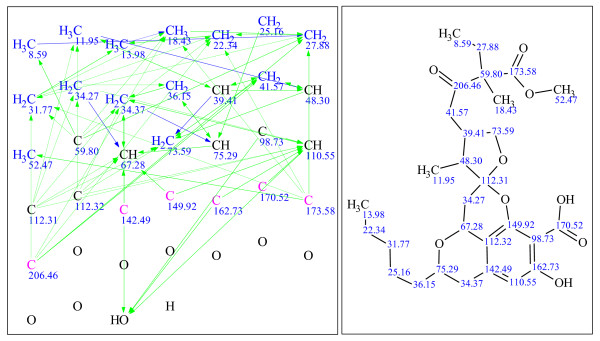
**Example 4: a single-blind challenge **[16]** elucidated using StrucEluc version 9**.

**Figure 13:**
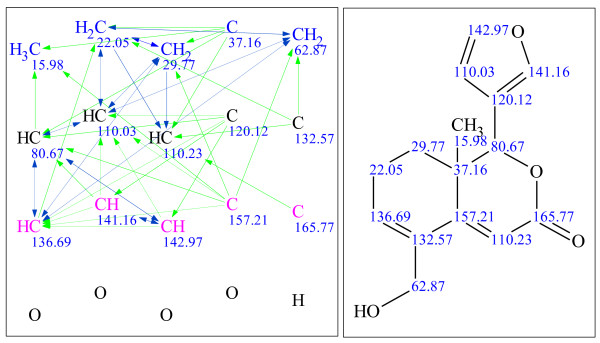
**Example 5: a single-blind challenge **[25]** elucidated using version 9**.

**Figure 14:**
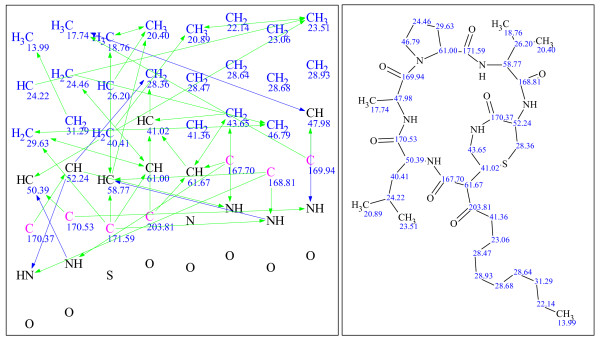
**Example 6: a single-blind challenge **[26]** elucidated using StrucEluc version 12**.

**Figure 15:**
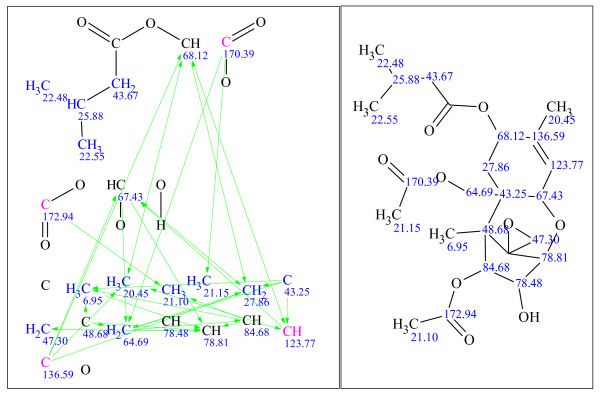
**Example 7: a single-blind challenge **[27]** elucidated using StrucEluc version 12**.

**Figure 16:**
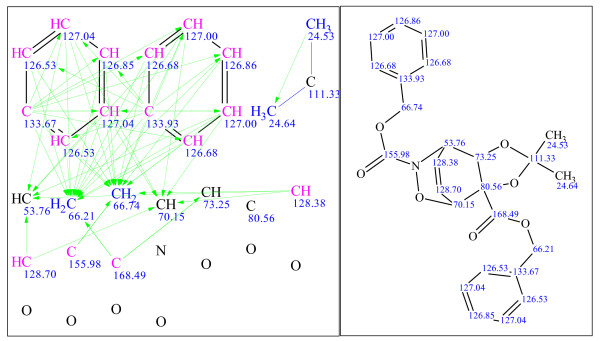
**Example 8: a single-blind challenge **[28]** elucidated using StrucEluc version 12**.

Tables 2, 3 and 4 summarize the results of eight single-blind challenges presented in detail in Figures 9, 10, 11, 12, 13, 14, 15 and 16. These test sets examine the elucidation of chemical structures varying in mass from 190 to 721 Da. The majority of challenges could be solved using typical data extracted from^ 1^H, ^1^H-^13^C HSQC and HMBC NMR. Multiplicity-edited HSQC data were used when available and were obviously preferred. In some trials, the data from the^ 1^H-^1^H COSY, TOCSY, NOESY and ROESY experiments were not required to solve the unknown. In some cases, the data from these experiments reduced the generation time from hours to minutes and assisted in the final stage of verifying the consistency for the final candidate. In one case, the submitter supplied spectral data in a table form, which was manually entered.

**Table 2:** Results of 8 single-blind trials.

Computer-assisted	Molecular Formula	MW (Da)	RDBE
**Example 1**	C_10_H_10_O_2_N_2_	190.2	7
**Example 2**	C_13_H_10_O_5_	246.2	9
**Example 3**	C_31_H_47_NO_11_	609.7	9
**Example 4**	C_30_H_42_O_9_	532.6	10
**Example 5**	C_15_H_16_O_4_	260.3	8
**Example 6**	C_36_H_60_N_6_O_7_S	721.0	10
**Example 7**	C_24_H_34_O_9_	465.5	8
**Example 8**	C_25_H_25_NO_7_	451.5	13

Molecular formulae, molecular weights and RDBE values of compounds.

**Table 3:** Results of 8 single-blind trials.

	1D NMR Data	2D NMR Data (Total Correlations/Ambiguous Correlations)	Data not used
**Example 1**	^1^H, ^13^C	HSQC, HMBC (10/2)	COSY
**Example 2**	^1^H, ^13^C, DEPT135	HSQC, HMBC (20/5)	COSY
**Example 3**	^1^H, ^13^C, DEPT135	HETCOR, HMBC(86/5), COSY (31/4)	ROESY
**Example 4**	^1^H, ^13^C	HSQC, HMBC(74/17), COSY (14/1)	-
**Example 5**	^1^H, ^13^C	HSQC, HMBC (23/4), COSY (25/12)	-
**Example 6**	^1^H, ^13^C	HSQC-DEPT, HMBC(67/0), COSY (31/0)	NOESY
**Example 7**	^1^H	HSQC, HMBC(47/0)	COSY, TOCSY
**Example 8**	^1^H, ^13^C	HSQC, HMBC(20/14), COSY(2/2)	-

Spectral data of compounds.

**Table 4:** Results of 8 single-blind trials.

	Position of Accepted Candidate	Number of Structures Generated	Spectral Processing Time (min.)	Generation Time (min.)	d^13^C HOSE (ppm)	stdd 13C (ppm)
**Example 1**	1	389	135	< 1	2.76	3.30
**Example 2**	1	50	Tabular	< 1	1.73	2.23
**Example 3**	1	9339	180	30	2.10	2.74
**Example 4**	1	388	180	6	2.20	3.05
**Example 5**	1	116	60	< 1	1.44	2.52
**Example 6**	1	9872	240	8	1.02	1.91
**Example 7**	1	127	120	< 1	0.84	1.07
**Example 8**	1	3224	120	60	1.82	2.08

Numerical data. In most cases, raw experimental NMR data files were submitted. For example 2, the processed data were submitted in the form of a table.

Example 3 exhibits a large number of candidates and a long generation time due to the high number of heteroatoms, 12, without any correlating NMR data, a number of atoms without defined hybridization states and a number of ambiguous correlations. These observations have been discussed previously [5,12,14].

It should be noted that the references for the publications listed beside Figures 9, 10, 11, 12, 13, 14, 15 and 16 were obtained from the submitter after the elucidation was performed, and presented herein as a source of spectral information. A number of publications have already reported the use of StrucEluc for the purpose of validating their proposed structure [15,16].

### 4. Handling Spectral Data

**Figure 17:**
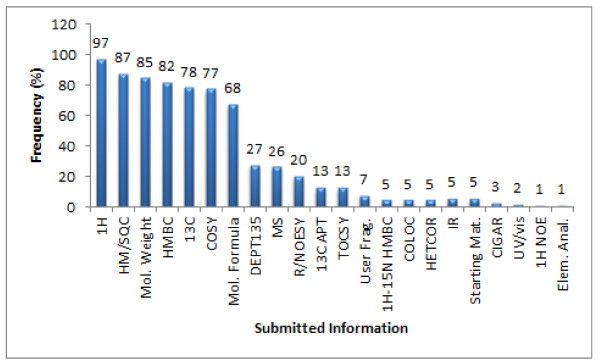
**A summary of the types of experiments submitted for all 112 challenges reported in this work**. Challenges that were rejected due to poor quality data are included.

The bar graph in Figure 17 summarizes the various types of datasets received during this research to examine the performance of the StrucEluc CASE program. For most of the challenges, we received the minimal required data as dictated by the guidelines of the challenge (see Experimental section for more details). A molecular weight, molecular formula, mass spectrum (MS), user fragment information and/or starting material was provided for about 85, 68, 26, 7, and 5% of the challenges, respectively. In three cases no information was provided regarding MF, MW, MS, user fragments or starting materials. All three challenges were nevertheless solved and subsequently verified by the submitters. Two cases were solved through a library search using the ^13^C chemical shifts. In the third case, an *sp *carbon was suggested by the software based on a^ 13^C chemical shift present in the spectrum; the submitter had not considered this option.

**Figure 18:**
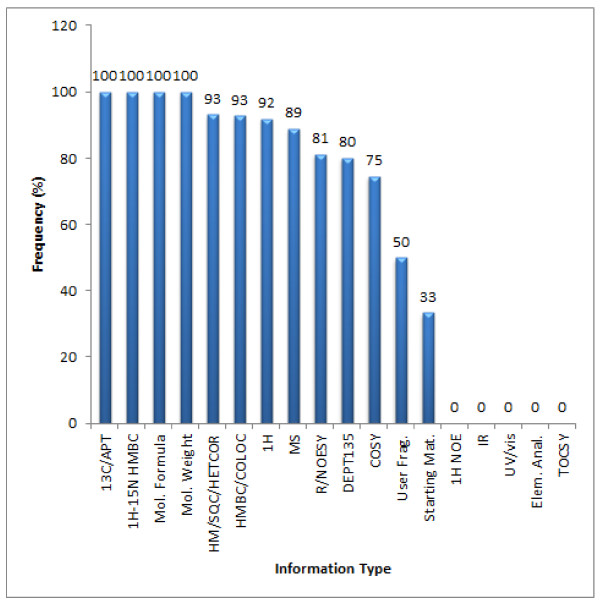
**A comparison between the types of experiments available and the usage frequency for the 100 Double and Single Agreement challenges**.

Figure 18 summarizes the experiments used to perform the elucidations using StrucEluc. In challenges where spectral data such as^ 13^C NMR and ^1^H-^15^N HMBC were available those data were utilized in all cases. In complex challenges that produced a large number of candidate structures, information regarding a fragment or starting material was helpful in reducing the generation time by establishing a portion of the structure and thereby reducing the number of potential candidates. This has been discussed in detail elsewhere [12,17].

Other experiments such as^ 1^H-^1^H TOCSY, NOE difference, UV/Vis and IR spectra were not used during the CASE elucidation process. Nevertheless the data were not necessarily superfluous but could still be utilized for candidate verification purposes. In those examples where a ^13^C chemical shift search was deemed to be successful, the^ 1^H NMR spectra were not required. This equated to 8% of the cases.

**Figure 19:**
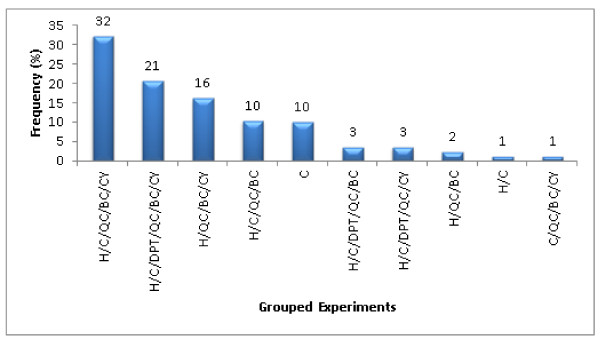
**The minimum set of spectral data used for the 100 Double and Single Agreement challenges**. Legend: H = ^1^H NMR, C = ^13^C NMR/APT/Pendant, DPT = ^13^C DEPT135, QC = ^1^H-^13^C HSQC/HMQC/HETCOR/HSQC-DEPT/HSQC-TOCSY, BC = ^1^H-^13^C/^1^H-^15^N HMBC/COLOC/CIGAR, CY = ^1^H-^1^H COSY, TY = ^1^H-^1^H TOCSY.

As a result of the analyses reported in this work it was possible to determine what pieces of spectral data were required to perform a computer-assisted structure elucidation and what data could be ignored without loss of fidelity in the results. This type of information can be useful in future experimental design for gathering data for an unknown. Figure 19 summarizes the minimal sets of spectral data employed in a challenge. The combination of spectral ^1^H/^13^C/HMQC/HMBC/COSY data were used in 33% of the challenges while only 15% represented the^ 1^H/HMQC/HMBC combination. In most challenges, long-range heteronuclear 2D NMR data were useful in reducing the number of potential candidates. When there were more types of data included in a dataset associated with a submitted challenge then more time was required for standard spectral processing of these additional data (i.e. Fourier transformation, phasing, peak-picking, assessing impurities, *etc*.).

Two key parameters representing an optimal CASE system are: 1) the time required to perform a successful elucidation relative to the time it would consume to perform the analysis manually and 2) the diverse range of candidates that can be investigated that would not be feasible if the analysis was attempted manually.

### 5. Categorizing the Candidates

**Figure 20:**
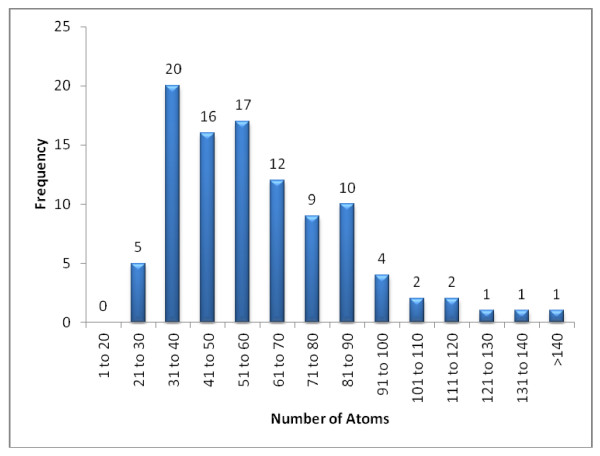
**The distribution of structures relative to the number skeletal atoms across 100 Double and Single Agreement challenges**.

The histogram in Figure 20 represents the distribution of structures relative to the number of skeletal atoms. A large portion of the compounds are within 31 to 90 atoms. Previous work by Elyashberg *et al. *[14] focused on the range of 20 to 50 skeletal atoms and had only 2 examples over 80 atoms. The elucidations performed in this work included over 20 challenges for unknowns containing over 80 atoms.

All unknowns were organic compounds typically containing C, H, O and N but also included atoms such as S, Br, Cl, F, and Na. Additional file 1 details the complexity of the molecular formulae. The challenges become more complex when N and S atoms in particular are present as these atoms can exist in multiple valence states and thus increase the number of potential candidates to be considered [1].

**Figure 21:**
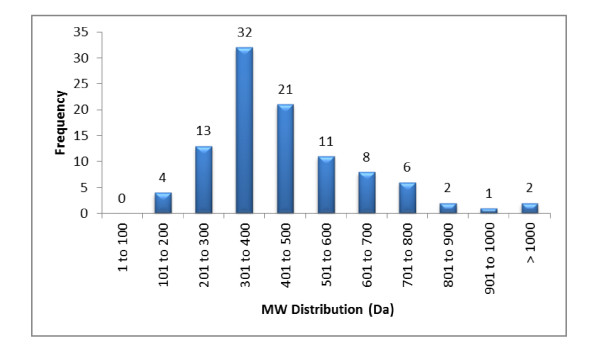
**The distribution of structures relative to the molecular weight across 100 Double and Single Agreement challenges**.

Figure 21 relates the distribution of the molecular weights across the frequency of each challenge. The candidates range from the smallest challenge at 149 Da to the biggest at 1256 Da with the average hovering around 419 Da.

The number of heavy atoms (excluding hydrogen atoms) contained within a MF ranges varies mainly from 10 to 90 atoms. The total number of heteroatoms range from 1 to 26. The Ring and Double Bond Equivalence (RDBE) ranges from 1 to 35. As these structure properties increase in number then the elucidation becomes more complex. In general of course, a higher mass relates to more atoms and the spectral data will be more challenging to interpret. This is a generality as clearly a high mass compound can have a simple spectrum: consider the buckminsterfullerene, C_60_, that has a single peak in the ^13^C NMR spectrum but the structural interpretation of the peak was not a simple problem. It is important to note that very large complex molecules can be elucidated quickly if they are rich in hydrogen atoms as the number of 2D NMR correlations will be high and, assuming there is not too much overlap elucidation may in fact be rather simple. An increase in the number of heteroatoms is demonstrated in the candidate structure as more combinations of positioning of the atoms. Higher RDBE values lead to complex ring systems and/or an abundance of quaternary carbon atoms and a deficiency in protons.

There are numerous attributes of complexity for the elucidation of an unknown using a CASE system. It is certainly not the complexity of a molecule to the human eye as many complex structures can be elucidated very efficiently by a CASE program that might initially seem intractable based on visual inspection. The degree of complexity is affected by the number of protons in the molecule, from which single and multi-bond correlations are generated in the 2D NMR spectra. A deficit of hydrogen atoms causes the greatest challenge as the number of direct and long-range correlations to use in the CASE analysis will be reduced. The level of ambiguity in terms of the quantity, diversity and nonstandard lengths of the long-range correlations is a major challenge [1,17]. A large number of candidates and a large generation time results from the interpretation and analysis of data complicated by these issues.

The complexity of the problem is further compounded by the presence of mixed heteroatoms (excluding C atoms), the presence of a salt and molecular symmetry. In 50% of the challenges the molecular formula included mixed heteroatoms. If the unknown contains mixed heteroatoms with an exchangeable proton such as OH and NH, the number of possibilities increases since an exchangeable proton can exist on either the oxygen or nitrogen atoms. If for instance there are two X-H bonds and the molecule contains two oxygen and two nitrogen atoms then the following combinations are possible: OH/OH, NH/NH or OH/NH. It is important to note that IR and Raman data can assist in distinguishing NH and OH groups. Over half the challenges consisted of mixed heteroatoms.

For 7% of the challenges, the submitted data corresponded to a sodium salt. As with the mixed heteroatoms, the number of sites of ionization and association with the sodium ion increases the potential candidates for elucidation of an unknown.

With 6% of the challenges exhibiting some form of structural symmetry, such as an inversion centre or a C_2 _axis, there is a higher incidence of coincident chemical shifts. The increase in ambiguity results in longer structure generation time. The problem of generating symmetric structures has been partly solved within the software recently [9] and we expect that symmetry will soon be used to facilitate the acceleration of structure generation.

StrucEluc attempts to generate a set of candidate structures consistent with the data. In many cases a pool of candidates is generated and a rank-ordering of the candidates in terms of their agreement with the experimental data is required in order to simplify user review. As discussed in detail elsewhere [1,17] a number of approaches are available including the comparison of experimental with predicted NMR spectra as well as comparison with mass spectral fragmentation data. The candidates can be ranked, for example, by the deviation between the experimental^ 13^C chemical shifts and the ^13^C shifts predicted using incremental and artificial neural network algorithms [2], as well as a HOSE code [18] based approach [1,17]. A deviation closer to zero signifies a better correspondence. Chemical shifts can be generated for ^1^H, ^13^C, ^15^N, ^31^P and^19^F nuclei using various algorithmic approaches and rank-ordering can be performed based on each of the predicted nuclei as well as by favored algorithm. The reader is encouraged to read the references [17,19] for details and examples.

The average ^13^C deviation for the top ranked structures is 2.2 ppm with a standard deviation of 3.1 ppm. ^13^C NMR shift prediction is chosen for the primary ranking as the predictions are less affected by solvent than^ 1^H NMR predictions. Based on the results of this work we have adjusted our benchmark deviations for future elucidations when separating good candidates from poor ones.

For Table 5, the average structure rank includes two challenges where the correct structures were ranked at positions 28 and 80. In both cases, the lists of candidates were very close in the ^13^C deviation and the submitters did not consider the proposed structure listed in the first position. Excluding these two challenges, the average structure ranking lists the correct structure in first place.

**Table 5:** The structure ranking for the 100 Double and Single Agreement challenges based on comparison of experimental versus predicted shifts.

	Structure Rank	^13^C Deviation (ppm)	^13^C STDD (ppm)
**Minimum**	1	0.04	1.21
**Maximum**	80	2.77	3.33
**Average**	2.5	2.17	3.18

The^ 13^C deviation is an average of all differences between the experimental and predicted^ 13^C chemical shifts whereas STDD is the standard deviation between all the experimental and predicted ^13^C chemical shifts.

### 6. Dealing with the Problem of Molecular Symmetry

**Figure 22:**
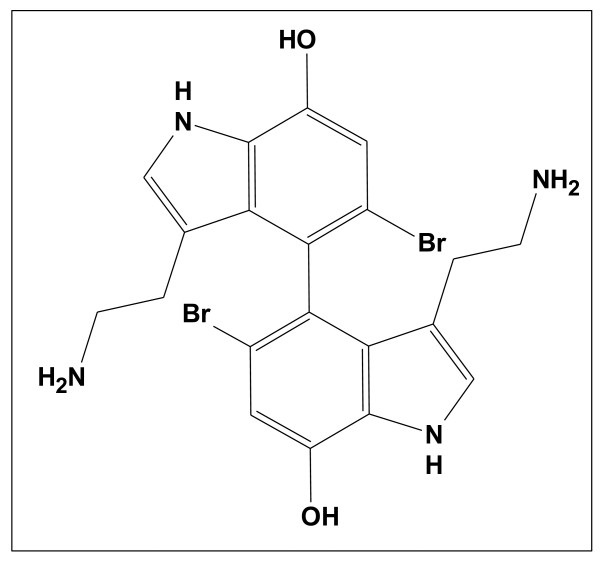
**Dendridine A, a bis-indole alkaloid from a marine sponge *Dictyodendrilla s*pecies (C_20_H_20_Br_2_N_4_O_2_) that exhibits a C_2 _axis is shown **[20].

Version to version StrucEluc has continued to be incrementally improved to accommodate the nuances of complex and challenging data and experiences obtained from solving hundreds of problems. The application of the software over the decade since initial development has helped to characterize a wide variety of analyzed structures and associated spectral data. While this publication cannot exhaustively examine the incremental design and algorithm changes which have occurred from version to version, and for that the reader is referred to our myriad of publications and review articles. However, an example of the impact of one algorithm enhancement on the performance of the software does warrant mention. For many years it was observed that the algorithm for structure generation from 2D NMR data failed to solve a problem in a reasonable time if the molecule under investigation (even of a modest size) was symmetric. To overcome this difficulty, the algorithm was reworked in such a manner to detect the presence of molecular symmetry from a logical analysis of the NMR spectral data and to perform structure generation taking into account the molecular symmetry. During the process of algorithm improvement the performance was continuously tested using a particular set of structures. One of these representative compounds uses the experimental data borrowed from the work of Tsuda *et al. *[20]. The structure for Dendridine A (C_20_H_20_Br_2_N_4_O_2_) exhibits a C_2 _axis (see Figure 22).

**Figure 23:**
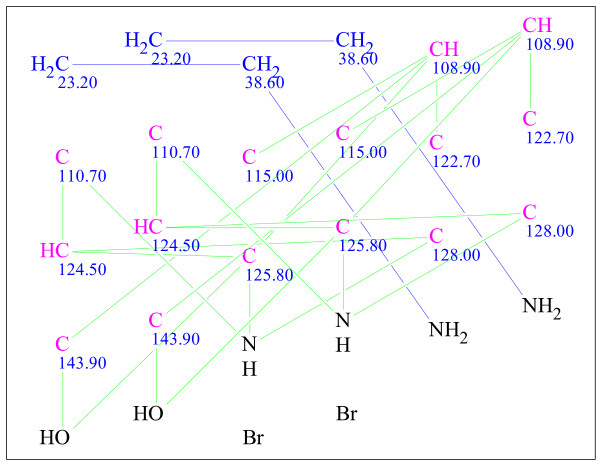
**The MCD for Dendridine A**. The blue and green lines represent the connectivities extracted from a^ 1^H-^1^H COSY and^ 1^H-^13^C HMBC spectra, respectively. The carbon atom colors dictate the hybridization state of the atom: blue and pink represent *sp^3 ^*and *sp^2^*, respectively.

Figure 23 shows that the 2D NMR data produced ^1^H-^1^H COSY (blue lines) and ^1^H-^13^C HMBC (green lines) correlations and only one pair of CH_2 _groups were defined by the program as having no heteroatom neighbours. This indicates that all other carbon atoms may be connected with N, O or Br atoms, and it can be concluded that a great number of structures may appear during the structure generation process.

The step-by-step progress in improvements regarding the performance of StrucEluc in dealing with symmetry is illustrated in Table 6. The table shows the initial difficulty of generating symmetric molecules with the version available in 2005 and the incremental improvement in the results as a result of adjusting the algorithm in 2006. Further improvements in performance between StrucEluc versions reduced both the output file size and the time associated with structure generation. Many algorithmic improvements were introduced over the lifetime of the software but such examples have become very useful for emphasizing the impact of particular algorithmic enhancements as well as helping to isolate classes of structural challenges requiring focused efforts. The details regarding the symmetry handling will be discussed in detail in a separate publication.

**Table 6:** Version to version changes in performance as a result of attempting to deal with structure symmetry issues using StrucEluc.

Version	Number of Structures Generated	Generation time (min.)	Position of Accepted Candidate	Process Status
8.0	25	131	Not Present	Aborted
8.1	132	77	Not Present	Aborted
8.2	1294	1049	Not Present	Aborted
8.3	-	2640	Not Present	Aborted
9.0	3964	235	1	Completed
9.1	4012	82	1	Completed
9.2	4012	80	1	Completed
9.3	4012	20	1	Completed
9.4	10264	17	1	Completed
12.1	34	10	1	Completed

The Process Status is listed as aborted if the structure generation required too long time for completion. The process was streamlined from version 9 to 12.

### 7. Spectral Purity

Spectral purity is an important criterion for a successful and relatively pain-free elucidation. Datasets that exhibit poor signal-to-noise, poor signal resolution, unexpected impurities, mixtures and/or artefacts tend to produce longer generation times, higher numbers of candidates, and in some cases, prevent any sensible candidates [5]. Since submitters vary in their laboratory procedures in regards to how samples are prepared and how the NMR data is acquired, a range of datasets varying in spectral purity were received. Datasets deemed to be of too low a quality were rejected and requests for better data collection by the client were issued.

**Figure 24:**
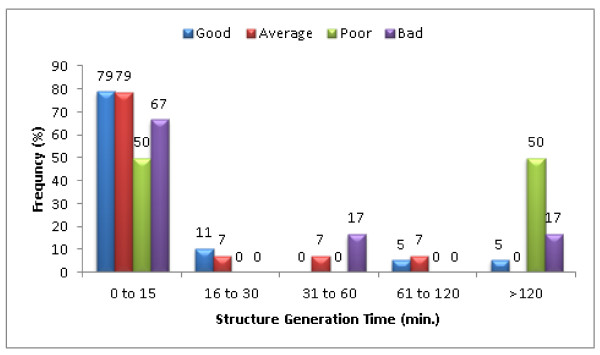
**Distribution of the structure generation time (minutes) relative to the overall NMR spectral purity across the blind trials classified as Double and Single Agreement challenges**. The datasets were judged based on the number of incidences of ambiguous assignments across all the NMR experiments. The number of incidences of ambiguous assignment and superfluous signals for good, average, poor and bad data are < 5, 5-10, 11-20 and > 20, respectively.

Figure 24 shows the distribution of structure generation time (in minutes) relative to the overall NMR spectral purity judged by the number of incidences of ambiguous assignment and superfluous signals. The number of incidences of ambiguous assignment and superfluous signals for good, average, poor and bad data are < 5, 5-10, 11-20 and > 20 ppm, respectively. Over 50% of the challenges generated a pool of candidate structures in less than 15 minutes. For challenges taking over 120 minutes, 5% were of good quality. These challenges had few long-range NMR correlations and needed more time to generate the candidates.

**Figure 25:**
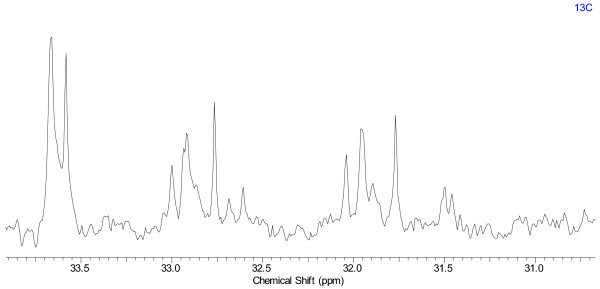
**An expansion of a ^13^C NMR spectrum submitted for analysis**. The data was collected in CDCl_3 _on a 600 MHz instrument, ns = 2048, points count = 16384, aq = 0.4555s, pulse sequence = zgpg30, sw = 35970.13 Hz, T = 25.0°C.

Figure 25 illustrates an example of a^ 13^C NMR spectrum submitted for analysis. The sample represents a mixture of unknown composition with over eighty potential signals. The uncertainty from the irregular line shapes adds to the complexity of analysis. The peak picking process became an exercise of trial-and-error and was halted.

### Experimental

The submitter was requested to submit a minimum data series, and if chosen, could provide additional data [8]. As part of the submission process, a structure, if known, should not be presented until after the analysis was complete. The challenge was not limited to spectral data of known materials but also invited unknowns to be submitted. The challenge was limited to two per submitter. The list presented to the submitter is shown below.

Minimum Required Data:

^1^H-^13^C HMQC, HSQC, HSQC-DEPT, HSQC-TOCSY, or HETCOR

^1^H-^13^C HMBC, long-range HETCOR, or LR HETCOR variants

^1^H NMR Survey Spectrum

^1^H-^1^H: COSY, DQF-COSY, TOCSY with short (< = 30 ms mixing time)

Molecular formula, mass spectrum or molecular weight (MF is preferred)

Additional Data:

^13^C NMR Simple Survey or tabularized^ 13^C shifts, multiplicities, and intensities

For nitrogen-containing compounds:^ 1^H-^15^N HMQC or HSQC

For nitrogen-containing compounds: ^1^H-^15^N HMBC

IR spectrum or tabularized data

MS spectrum or table of peaks

Other general information, such as starting materials, related molecules (e.g., parent family of natural products), derivatives, metabolites, etc.

TOCSY (any mixing time)

XCORFE and other long-range Heteronuclear correlation experiments

INADEQUATE

DEPT, APT

NOESY, NOE-Difference data, ROESY (depending on mixing scheme)

Information of any impurities present in the data

## Conclusion

The penultimate test for a CASE application is through a set of blind trials. In this approach a submitter withholds the information on the structure so as not to bias the software operator. This is a single-blind trial. Double-blind trials serve the ultimate test and characterize the situation where the structure is unknown to both parties. We have reported a review of the analysis of 112 unique challenges submitted as either single or double-blind trials and the performance characteristics of the CASE system ACD/Structure Elucidator. Unfortunately the details of many of these elucidation studies have not been reported as the majority of the elucidations were performed under non-disclosure agreements. The software and underlying algorithms described in this work have been shown to offer excellent performance throughout these trials.

Our studies have demonstrated that the most ideal data sets for analysis include a single molecular formula (likely extracted from a high resolution mass spectrum), a pure spectrum (no complexities in the spectrum due to the presence of contaminants, tautomers, restricted rotation, *etc*.), a sufficient number of heteronuclear correlations to fully define the molecular skeleton and a minimal number of long-range correlations spanning > 3 bonds. While these are the ideals, iterative development of the software allows even these limitations to be handled. The results are sufficiently encouraging to suggest that CASE systems should become general utility tools for chemists to accelerate the identification of compounds with increased probability of success.

## Abbreviations

CASE: Computer-Assisted Structure Elucidation; NMR: Nuclear Magnetic Resonance; COSY: COrrelation SpectroscopY; HSQC: Heteronuclear Single Quantum Correlation; HMBC: Heteronuclear Multiple-Bond Correlation; MF: Molecular Formula; MW: Molecular Weight; SM: Starting Material or derivatives; MS: Mass Spectrometry; EA: Elemental Analysis; UV: UltraViolet; IR: InfraRed; RDBE: Ring and Double-Bond Equivalence; STDD: STanDard Deviation; MCD: Molecular Connectivity Diagram; RDBE: Ring Double Bond Equivalence

## Competing interests

The Structure Elucidator software program discussed in this publication is a commercial software product marketed by Advanced Chemistry Development (ACD/Labs). All authors are employees, ex-employees or collaborators of ACD/Labs.

## Authors' contributions

MEE has been involved with the development of the Structure Elucidator software package for over a decade. AJW was the product manager for Structure Elucidator for over a decade during his employment with ACD/Labs and remains an active collaborator. KAB has been the project leader for Structure Elucidator since its inception. JCD was the trainer for Structure Elucidator and assisted with the challenges. AM was the trainer for Structure Elucidator and assisted with the challenges. All authors read and approved the final manuscript.

## Supplementary Material

Additional file 1 **Summary of the atom ranges, RDBE, MW and heteroatom count for the trials**. The data provided represent a summary of the ranges of composition, the ring and double-bond equivalence (RDBE), molecular weight (MW) and total number of heteroatoms for the trials. Click here for file
