# Supplementary material for: Blind trials of computer-assisted structure elucidation software
Source: J Cheminform. 2012 Feb 9;4:5. doi: 10.1186/1758-2946-4-5 (PMC3349476; doi:10.1186/1758-2946-4-5)
Supplement: Additional file 1 — Summary of the atom ranges, RDBE, MW and heteroatom count for the trials. The data provided represent a summary of the ranges of composition, the ring and double-bond equivalence (RDBE), molecular weight (MW) and total number of heteroatoms for the trials. [file 1758-2946-4-5-S1.DOC]

**Supplemental Information**

The SI Tables present the results of the ranking for the submitted challenges.

**SI Table 1**. Summary of the ranges of composition for the trials.

|  | **C** | **H** | **O** | **N** | **S** | **Br** | **Cl** | **F** | **Na** | **HCl** |
| --- | --- | --- | --- | --- | --- | --- | --- | --- | --- | --- |
| **Minimum** | 10 | 10 | 0 | 0 | 0 | 0 | 0 | 0 | 0 | 0 |
| **Maximum** | 60 | 74 | 22 | 12 | 3 | 1 | 2 | 6 | 1 | 1 |
| **Average** | 23.7 | 27.8 | 5.5 | 1.4 |  |  |  |  |  |  |

**SI Table 2**. Summary of the ranges for the ring and double-bond equivalence (RDBE), molecular weight (MW) and total number of heteroatoms.

|  | **RDBE** | **MW (Da)** | **Number of Heteroatoms** |
| --- | --- | --- | --- |
| **Minimum** | 1 | 156 | 1 |
| **Maximum** | 35 | 1256 | 26 |
| **Average** | 11 | 419 | 8 |
